# Isolation, Molecular Typing, and Antibiotic Susceptibility Testing of *Mycobacterium avium* Subspecies *hominissuis* From a Dog With Generalized Mycobacteriosis

**DOI:** 10.3389/fvets.2020.569966

**Published:** 2020-11-04

**Authors:** Cinzia Marianelli, Daniela Ape, Federica Rossi Mori

**Affiliations:** ^1^Department of Food Safety, Nutrition and Veterinary Public Health, Istituto Superiore di Sanità, Rome, Italy; ^2^Belli Lisi Studio Veterinario, Monterotondo, Italy

**Keywords:** *Mycobacterium avium* subsp. *hominissuis*, dog, antimicrobial susceptibility, disseminated infection, SNPs typing, MIRU-VNTR analysis

## Abstract

*Mycobacterium avium-intracellulare* complex infections are becoming an increasing concern in veterinary medicine because they affect livestock, wildlife, and companion animals. Here we describe the isolation, molecular typing, and antibiotic susceptibility testing of the causative agent of a rare case of generalized mycobacteriosis in a crossbred dog. Mycobacterial colonies were isolated from a popliteal lymph node aspirate sample and molecular typed by SNPs typing of the genes *gyrB* and *rpsA*, the 3′ region of the *hsp65* gene and the internal transcribed spacer (ITS), and MIRU-VNTR analysis. Colonies were also tested *in vitro* against the macrolide clarithromycin and other drugs, using a resazurin microdilution assay, in order to provide the most appropriate treatment for the dog. Results from SNPs typing of *gyrB* and ITS, as well as from MIRU-VNTR analysis suggested the isolation of a single strain of *M. avium* subsp. *hominissuis* (Mah). On the other hand, SNP typing of *rpsA* revealed DNA polymorphisms that led colonies to cluster into two groups. The presence of two distinct strains of Mah has been assumed. All colonies, regardless of the nucleotide sequence of *rpsA*, were found to be sensitive to all of the drugs tested except for ethambutol. Although the therapy administered was adequate, the dog's overall clinical status worsened progressively and the animal died 8 months later. In conclusion, we report on the isolation of Mah from a dog with generalized mycobacteriosis.

## Introduction

*Mycobacterium avium-intracellulare* complex (MAC) infections are becoming an increasing concern in veterinary medicine because they occur in a wide range of animals, including domestic animals (1), ruminant and non-ruminant wildlife (2), and companion animals (3, 4). MAC includes two main species: *M. avium* and *M. intracellulare* (5). *M. avium*, the most clinically significant in both humans and animals, comprises four subspecies: *M. avium* subsp. *hominissuis* (Mah), *M. avium* subsp. *paratuberculosis* (Map), *M. avium* subsp. *avium* (Maa), and *M. avium* subsp. *silvaticum* (5).

MAC infections are rare in dogs. Although only a small number of cases have been published, certain breeds, such as basset hounds and miniature schnauzers, seem to be at higher risk (3, 4, 6, 7). MAC infections may cause granulomatous diseases, which range from localized granulomas to disseminated diseases. Disseminated MAC infections are sporadic and are associated with a poor prognosis (4, 6). Many cases of canine MAC infection discussed in the literature have not been typed at subspecies level (4, 8). We therefore have no accurate data concerning the pathogenicity of MAC subspecies in these companion animals. However, in a few cases, molecular typing at subspecies level has been carried out and Mah has proved to be the most frequent isolate (6, 7, 9).

Several molecular typing methods have been developed to differentiate between *M. avium* subspecies and strains, such as single nucleotide polymorphism (SNP) typing and mini-satellite sequence analysis—mycobacterial interspersed repetitive-unit variable-number tandem repeats (MIRU-VNTR) (5). SNP typing takes advantage of nucleotide changes in specific single-copy genes—i.e., *gyrA, gyrB, groEL1, sodA, rpsA*, and *hsp65*—(10–12) or chromosomal loci—i.e., 16S−23S rDNA internal transcribed spacer (ITS)—(13) to use them as biomarkers for subspecies differentiation. MIRU-VNTR analysis, on the other hand, uses mini-satellite sequences (tandem repeats of 10–100 nucleotides) dispersed at multiple loci in the bacterial genome. MIRU-VNTR represents one of the most plastic elements within mycobacterial genomes (14, 15).

We report the results of the investigation of a rare case of disseminated mycobacteriosis in a crossbred dog. Our aims were (1) to isolate and molecular type the causative agent, and (2) to determine antibiotic susceptibility *in vitro* so as to ensure the most appropriate antimicrobial therapy for the dog.

## Materials and Methods

### Case Description and Sample Collection

A mycobacterial infection was suspected by one of the authors (FRM) in a 3 year-old crossbred female dog presenting with fever, lack of appetite, lymphadenomegaly, enlarged spleen, abdominal effusions, bowel wall thickening. Fine-needle aspiration biopsies of the popliteal and mesenteric lymph nodes, and of the spleen and abdominal fluids, were carried out under general anesthesia. Samples were stained using May-Grünwald-Giemsa stain and the routine Ziehl–Neelsen method. Based on cytology results (Supplementary Figure 1), the diagnosis was confirmed. A therapy based on enrofloxacin 10 mg (kg body weight)^−1^ every 24 h *per os* was prescribed. After a period of initial improvement (2 months), the dog continued to deteriorate. A second fine-needle aspirate sample of about 500 μl was collected from the popliteal lymph node and sent to the Istituto Superiore di Sanità in Rome for further investigation. Azithromycin 10 mg (kg body weight)^−1^ every 24 h *per os* and rifampicin 10 mg (kg body weight)^−1^ every 12 h *per os* were added to the treatment. Twenty days later, rifampicin was discontinued because it caused diarrhea as a side effect, and enrofloxacin and azithromycin were continued.

### Isolation of the Pathogen and Molecular Identification of Species

The popliteal lymph node aspirate sample was centrifuged for 10 s at 13,000 × g and the supernatant discarded. The pellet was suspended in 500 μl of sterile PBS. One hundred microlitres of this suspension was inoculated in Middlebrook 7H9 broth (Biolife, Italy) with 10% oleic acid albumin dextrose complex (OACD) (Becton, Dickinson and Company, USA). In order to isolate the organisms, a 10 μl disposable loop was used to streak the suspension on Middlebrook 7H11 agar (Biolife, Italy) with 10% OACD enrichment. Both the broth culture and agar plate were then incubated in CO_2_ for 3 weeks at 37°C for bacteriological examination.

After incubation, four colonies were randomly picked from the agar plate and subcultured in 1 ml supplemented Middlebrook 7H9 broth for 1 week. DNA was then extracted from both the primary broth culture and the four colony-derived subcultures using a commercial kit (InstaGene Matrix; Bio-Rad Laboratories, Italy) following the manufacturer's instructions.

Two μl of DNA from each sample was used as a template for the multiplex PCR assay previously described (16), with some modifications (also as previously described) (17), in order to detect and identify members of the genus *Mycobacterium*, and to differentiate between members of the *M. tuberculosis* complex (MTC), *M. avium* and *M. intracellulare*. Reactions were performed in a total volume of 50 μl using MyTaq Red DNA Polymerase (Bioline, London, UK) following the manufacturer's instructions and the cycling conditions previously reported (17). Field isolates of *M. bovis* and *M. avium* from our collection were used as controls. PCR samples positive for *M. avium* were examined further for subspecies identification.

### Molecular Identification of *M. avium* Subspecies

#### SNPs Typing

The four colonies, as well as the primary broth culture, positive for *M. avium* were typed at subspecies level. Both a 353 bp fragment of the *gyrB* gene encoding the DNA gyrase B and a 933 bp fragment of the *rpsA* gene encoding the ribosomal protein S1 were PCR amplified and sequenced as previously described (17).

On the basis of the results (the *rpsA* gene polymorphisms), 18 additional colonies were randomly picked from the agar plate and subcultured as described above, and DNA was then extracted. PCR amplification and sequencing of the partial genes *gyrB* and *rpsA* were performed to confirm the results. Colonies were then examined further: a 1059 bp fragment of the more variable 3′ region of the *hsp65* gene (3′*hsp65*) (12) and the complete 16S−23S rDNA internal transcribed spacer (ITS) (13), about 600 bp in length, were PCR-amplified. Reactions were performed in a total volume of 50 μl using MyTaq Red DNA Polymerase (Bioline) following the manufacturer's instructions. The cycling conditions for both PCR amplifications were: denaturation at 94°C for 5 min, followed by 35 cycles at 94°C for 20 s, 57°C for 30 s, and 72°C for 60 s, and a final extension cycle at 72°C for 7 min. All PCR products were purified and then sequenced using the same PCR primers.

All primers used for both PCR amplifications and sequencing are shown in Table 1. Sequences were analyzed using ABI Prism SeqScape software, version 2.0 (Applied Biosystems). Field isolates of Maa and Map from our collection were used as controls. The consensus sequences generated by matching forward and reverse reads and removing PCR primers have been compared to sequence databases and identified using the BLAST tool (https://blast.ncbi.nlm.nih.gov/Blast.cgi).

**Table 1:** Primers used for PCR and sequencing.

**Gene**	**Forward and reverse primers**	**Size (bp)**	**Reference**
*gyrB*	F 5′- GCAGACGCCAAAGTCATTGT -3′ R 5′- TCGAACTCGTCGTGAATCCC -3′	353	(17)
*rpsA*	F 5′- CTTCTCGAATCCCTCGAGCC -3′ R 5′- CGCCTGATCCTGTCCAAGAA -3′	933	(17)
3′*hsp65*	F 5′- CGGTTCGACAAGGGTTACAT -3′ R 5′- ACGGACTCAGAAGTCCATGC -3′	1059	(12)
ITS	F 5′- TTGTACACACCGCCCGTCA -3′ R 5′- TCTCGATGCCAAGGCATCCA -3′	~600	(13)

#### MIRU-VNTR Analysis

A total of six colonies (three for each different *rpsA* nucleotide sequence identified here) were PCR-amplified at 8 MIRU-VNTR loci—TR32, TR392, TRX3, TR25, TR3, TR7, TR10, and TR47—as previously described (15). Reactions were performed in a total volume of 50 μl using MyTaq Red DNA Polymerase (Bioline) following the manufacturer's instructions. One μl of dimethyl sulfoxide (Sigma) was added to each reaction (15). The cycling conditions for all PCR amplifications were: denaturation at 94°C for 5 min, followed by 40 cycles at 94°C for 30 s, 57°C for 30 s, and 72°C for 60 s, and a final extension cycle at 72°C for 7 min. To estimate exactly the amplicon sizes and thus determine the number of repeated units for each locus, all PCR products were purified and then sequenced using the same PCR primers. The Codon Code Aligner software 9.0.1 (CodonCode Corporation) was used for forward and reverse sequence assembly. MIRU-VNTR profiles were determined using the table showing the theoretical size of PCR products available at the MAC-INMV-SSR database website (http://mac-inmv.tours.inra.fr/index.php?p=fa_download) and then run against the freely accessible MAC-INMV-SSR database (http://mac-inmv.tours.inra.fr/index.php) (18) to identify the pathogen at subspecies level.

### Susceptibility Testing

Seven samples were tested: one from the primary broth culture, and six from single colony-derived subcultures (three for each different *rpsA* nucleotide sequence here identified) previously typed by MIRU-VNTR. The drug susceptibility testing was performed using the resazurin microtitre assay (REMA) according to the protocol previously described (17).

Samples were assessed for susceptibility to the drugs administered: the macrolide clarithromycin, which is the recommended class antibiotic for *in vitro* testing due to technical issues with azithromycin; ciprofloxacin, which is the active metabolite of enrofloxacin; and rifampicin (Sigma-Aldrich, UK). Sensitivity to five additional drugs was also tested: streptomycin, amikacin, doxycycline, ethambutol, and linezolid (Sigma-Aldrich, UK). Because standard laboratory guidelines for *in vitro* susceptibility testing for MAC—with the exception of clarithromycin and linezolid—are not available, tentative breakpoints from the literature (19–21) and the Clinical and Laboratory Standards Institute (CSLI) guidelines for Rapidly Growing Mycobacteria (22) were used. Further details, including the minimal inhibitory concentration (MIC) breakpoints used for each antimicrobial agent, are reported in Table 2.

**Table 2:** Antimicrobial agents tested and proposed MIC breakpoints for MAC.

**Antimicrobial agent**	**MIC breakpoints (μg ml**^****−1****^**)**			**References**
	**S**	**I**	**R**	
Clarithromycin^*^	≤16	32	≥64	(21)
Streptomycin	≤16	32	≥64	(19)
Amikacin	≤16	32	≥64	(18, 19)
Rifampicin	≤1	-	≥2	(21)
Ciprofloxacin	≤1	2	≥4	(21)
Doxycycline	≤1	8	≥16	(21)
Ethambutol	≤2	4	≥8	(20)
Linezolid^*^	≤8	16	≥32	(21)

*S, susceptible; I, intermediate; R, resistant*.

* *Standard laboratory guidelines for in vitro susceptibility testing for MAC isolates are only reported for clarithromycin (as primary agent) and linezolid (as secondary agent). Clarithromycin serves as a class drug for all newer macrolides, especially azithromycin. According to CLSI recommendations, clarithromycin was tested in Middlebrook 7H9 broth at pH 6.8*.

## Results

### Identification of the Mycobacterial Pathogen at Species and Subspecies Levels

Fine-needle aspiration (FNA) cytology of enlarged lymph nodes revealed numerous slender, rod-shaped, acid-fast organisms suggestive of *Mycobacterium*, located inside and outside the macrophages (Supplementary Figure 1). A mycobacterial infection was diagnosed.

Bacteria were isolated from the popliteal lymph node aspirate sample after 3 weeks of incubation. The streak plate showed the appearance of opaque colonies growing in a lawn of translucent colonies. Four colonies, including both opaque (*n* = 2) and translucent colonies (*n* = 2), were picked randomly and subcultured. Samples from the primary broth culture and the four colonies were subjected to molecular typing for species identification of the pathogen. A 1030 and a 180 bp amplification product indicative of the genus *Mycobacterium* and of *M. avium*, respectively, was obtained in all samples and a diagnosis of *M. avium* infection was made (Supplementary Figure 2). To type the pathogen at subspecies level, the four colonies were assessed by both SNP typing and MIRU-VNTR analysis.

All nucleotide sequences of *gyrB* amplicons from the four colony-derived subcultures (*n* = 4) and from the primary broth culture were identical [100% identity] Mah strain H87, *M. avium* 104 and *M. avium* SSM4139 (sequence ID: CP018363.1, CP000479.1, and DQ386879.1, respectively), available in the NCBI database. On the other hand, two different *rpsA* sequences carrying nucleotide variability at four sites (SNPs) were found: AAT → GAT at codon 391 (mutation N391D, SNP1), ACC → GCC at codon 754 (mutation T754A, SNP2), CAG → GAG at codon 760 (mutation Q760E, SNP3), and AAC → GAC at codon 784 (mutation N784D, SNP4). One colony-derived subculture carried all four SNPs (group A), while three colony-derived subcultures and the primary broth culture carried none of the above synonymous mutations (group B). BLAST results using an *rpsA* sequence from group A as query showed 100% identity with seven Mah strains available in the database (sequence ID: CP029332.1, CP018014.2, CP018020.2, LC079180.1, AP012555.1, CP016818.3, and CP009360.4). When *rpsA* sequences from group B were used as query, the closest match found in the database (100% identity) was only with *M. avium* 104 (sequence ID: CP000479.1). Although *M. avium* 104 subspecies is mentioned as “unknown” in the database, nowadays it is known to be an Mah strain.

Eighteen additional colonies, including both colonial morphology variants, were then picked randomly, subcultured and subjected to both *gyrB* and *rpsA* sequencing in order to confirm the results. All colonies had identical *gyrB* sequences to the previous ones. Sequencing of the partial r*psA* gene grouped the 18 colonies into group A (*n* = 5, 28%) and group B (*n* = 13, 72%). *RpsA* sequencing results are shown in Table 3. No association was found between the *rpsA* nucleotide sequence and the morphology of the colony (translucent or opaque).

**Table 3:** Nucleotide and amino acid changes in the *rpsA* gene of a total of 22 colonies (the initial four colonies and the subsequent 18 colonies).

**Group**	**No of** **colonies**	**Codon position**			
		**391**	**754**	**760**	**784**
A	6	GAT (D)	GCC (A)	GAG (E)	GAC (D)
B	16	AAT (N)	ACC (T)	CAG (Q)	AAC (N)

The colonies were then investigated at two additional polymorphic sites: 3′*hsp65* and ITS. Sequencing both 3′*hsp65* and ITS, as with *gyrB*, all colonies showed identical sequences within each target gene analyzed. The nucleotide sequences of 3′hsp65 were identical to *M. avium* 28,132 in the database (sequence ID: DQ284767.1), subspecies unknown, thus hampering accurate identification at subspecies level. Conversely, ITS nucleotide sequences were identical to six Mah strains in the database (sequence ID: AP020326.1, CP040250.1, CP035744.1, CP018363.1, CP000479.1, and AF410479.1).

We extended our analysis using MIRU-VNTR typing. All the colonies analyzed, regardless of the nucleotide sequence of *rpsA*, showed the same profile (2,4,2,2,1,1,2,8), which perfectly matched Mah INMV 51 in the MAC-INMV-SSR database. We therefore confirmed an Mah infection. The only genetic variability revealed here between colonies was therefore limited to the *rpsA* gene. Taken together, the results of this study suggested the presence of two distinct isolates of Mah in the animal.

### Drug Susceptibility Profile

**Figure 1:**
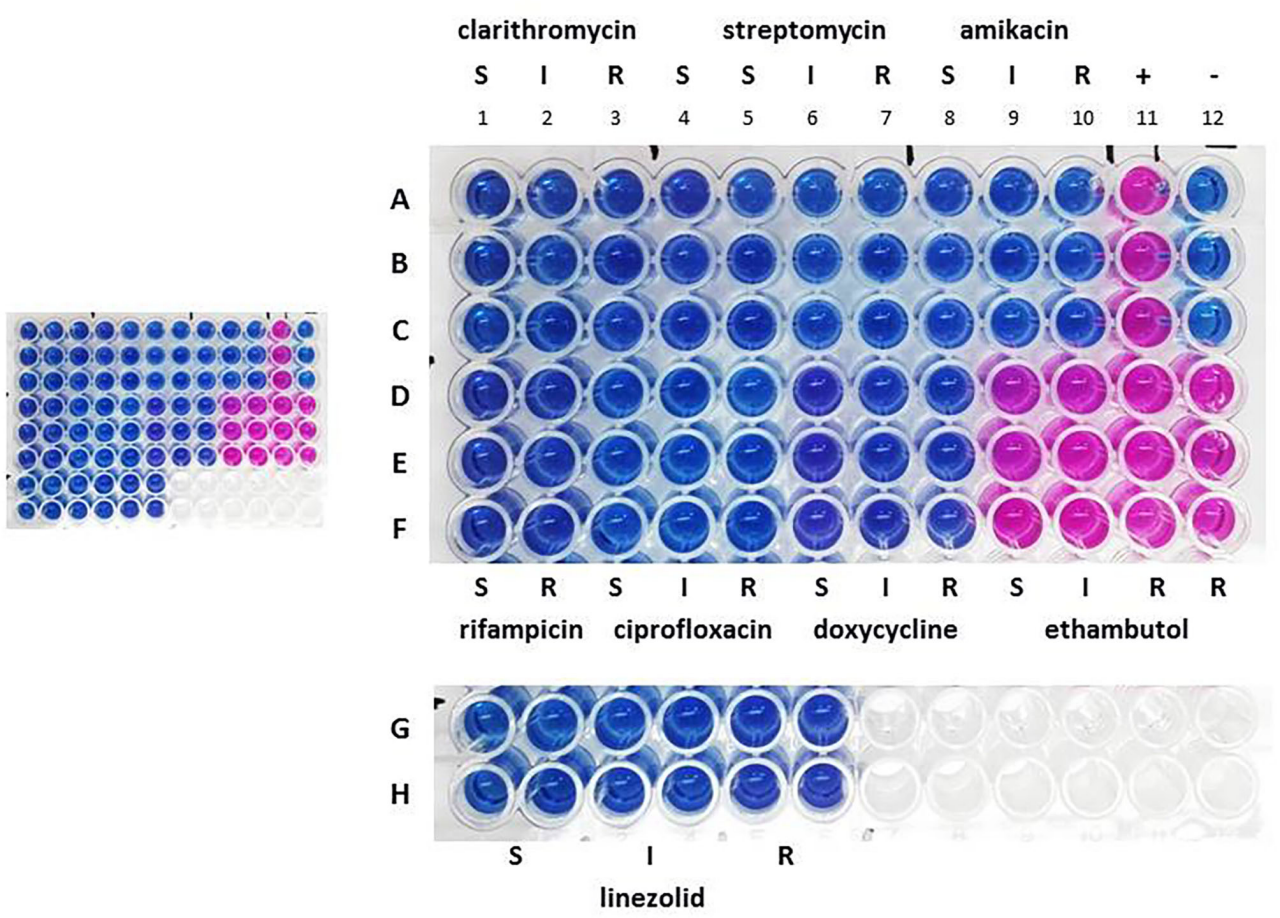
Drug susceptibility profile the primary broth culture. A–F: sample triplicates. G, H: sample quadruplicates. S, susceptible; I, intermediate; R, resistant. Lines A–C, columns 1–3: 16.0 (S), 32.0 (I), and 64.0 (R) μg ml^−1^ for clarithromycin; columns 4–7: 8.0 (S), 16.0 (S), 32.0 (I), and 64.0 (R) μg ml^−1^ for streptomycin; columns 8–10: 16.0 (S), 32.0 (I), and 64.0 (R) μg ml^−1^ for amikacin; column 11: positive control containing no drug (+); column 12: negative control containing uninoculated medium (–). Lines D–F, columns 1, 2: 1.0 (S) and 2.0 (R) μg ml^−1^ for rifampicin; columns 3–5: 1.0 (S), 2.0 (I), and 4.0 (R) μg ml^−1^ for ciprofloxacin; columns 6–8: 1.0 (S), 8.0 (I), 16.0 (R) μg ml^−1^ for doxycycline; columns 9–12: 2.0 (S), 4.0 (I), 8.0 (R), 16.0 (R) μg ml^−1^ for ethambutol. Lines G, H, columns 1, 2, 3, 4, 5, 6: 8.0 (S), 16.0 (I), 32.0 (R) μg ml^−1^ for linezolid.

Drug susceptibility testing was performed in order to ensure that the dog received the most appropriate therapy. We used REMA, a broth microdilution test method, and the results were available after 7 days of incubation. Identical drug susceptibility profiles were obtained by testing both the primary broth culture and six single colony-derived subcultures (three from group A and three form group B). Results are shown in Figure 1.

All isolates were found to be sensitive to clarithromycin, streptomycin, amikacin, rifampicin, ciprofloxacin, doxycycline and linezolid, but were resistant to ethambutol. The resazurin blue dye was reduced to pink at the resistant breakpoint for ethambutol (R, 8 μg ml^−1^) (Figure 1, column 11, lines D–F). Repeated tests confirmed these results.

The therapy based on enrofloxacin, azithromycin, and rifampicin was confirmed. The infection did not resolve and the dog died 8 months after the diagnosis.

## Discussion

We describe here a rare case of generalized mycobacteriosis in a crossbred dog where the results of an in-depth molecular investigation suggested an infection with two distinct isolates of Mah.

Opportunistic mycobacterial infections caused by MAC are rare in dogs and generally have non-specific clinical signs, making *ante mortem* diagnosis difficult. When the forms progress to generalized disease, the prognosis is poor (7). In those few cases of canine mycobacteriosis where the pathogen has been isolated and typed, Mah was the most common aetiological agent (6, 7, 9). Mah is an opportunistic environmental pathogen and is considered the clinically most important MAC member for both humans and animals (23). Soil and water are considered to be the natural reservoirs of the organisms. Drinking water and tap aerosols are thought to be the main sources of Mah infections (24).

It is likely that the dog acquired the MAC pathogen from the environment through ingestion or inhalation and the infection then spread to the lymph nodes and spleen. Unfortunately, when the diagnosis of a mycobacterial infection was made, the clinical signs in the animal were severe and the disease was generalized. We immediately performed all laboratory investigations to identify the pathogen and to select the most appropriate antibiotic therapy for the animal.

We isolated both translucent and opaque colonies from the lymph-node-aspirate sample culture. This result is consistent with a previous study where MAC strains have been shown to generate both opaque and transparent colonial variants when cultivated *in vitro* (25). Firstly, we typed the isolates at species level. All samples analyzed turned out to be *M. avium*. Then, we sequenced the most variable regions of *gyrB* and *rpsA* previously selected by our group (17), in order to type the colonies at subspecies level. We chose the *gyrB* and *rpsA* genes for our analysis because both genes have proven to be highly polymorphic and able to differentiate MAC subspecies (10, 11). Identical *gyrB* nucleotide sequences that perfectly matched Mah in the database were found when we analyzed the colonies. On the other hand, nucleotide sequence discrepancies were observed in the *rpsA* gene at four sites, which caused amino acid changes and divided the colonies in two groups—A and B. The additional colonies analyzed (*n* = 18) confirmed the *rpsA* gene polymorphisms detected in the previous colonies. The *rpsA* sequence of group A perfectly identified several Mah strains in the database. The *rpsA* sequence of group B, conversely, perfectly matched a different Mah strain in the database, namely *M. avium* 104. The RpsA protein—ribosomal protein S1, which is in the 30S ribosome subunit—is the target of pyrazinoic acid, the active form of the antituberculosis drug pyrazinamide. Mutations in the RpsA protein have been associated with a small number of cases of pyrazinamide resistance (26). Since pyrazinamide had never been administered to the dog, drug selection for mutant strains could not therefore have occurred. Moreover, it is highly unlikely that four synonymous mutations could have occurred in the *rpsA* gene by subculturing colonies just once—picked colonies were indeed analyzed after one passage—and in the absence of exogenous stress (i.e., pyrazinamide). These considerations, coupled with the fact that *rpsA* is a single-copy gene, led us to conclude that the *rpsA* gene polymorphisms observed reflected the existence of two distinct populations of Mah.

We then resorted to additional molecular typing approaches, such as the sequencing of 3′*hps65* (12)—the highly variable 3′ region of the *hps65*—and ITS (13)—the internal transcribed spacer between 16S rDNA and 23S rDNA, and 8-loci MIRU-VNTR analysis (15)—tandem repeat sequences dispersed at eight loci in the genome as source of genetic variability—in order to find additional polymorphisms which could confirm the presence of two different strains of Mah. As with *gyrB*, colonies from both groups A and B showed identical nucleotide sequences for 3′*hps65* and ITS, and identical MIRU-VNTR profiles. While 3′*hps65* sequences limited identification at species level, ITS sequences and MIRU-VNTR profiles permitted such identification, confirming the detection of Mah. It is widely known that the greater the number of loci included in the MIRU-VNTR typing, the higher the discriminatory power of the method becomes. Based on this assumption, it is likely that if a higher number of MIRU-VNTR loci had been analyzed, we would have been able to distinguish colonies from the two groups, as we did using *rpsA* sequencing.

We also performed drug susceptibility testing in order to ensure the most appropriate antimicrobial therapy for the dog. Standard laboratory guidelines for susceptibility testing of MAC are available only for clarithromycin as primary agent, and linezolid and moxifloxacin as secondary agents (22). Breakpoint concentrations for linezolid and moxifloxacin are also provided (22), although the clinical role of these compounds has not yet been established. With regard to the other agents used to treat MAC infections, no correlation between drug susceptibility testing and therapeutic outcome has been found (27), and tentative breakpoints from the literature have therefore been used in this study.

All tested samples revealed the same drug susceptibility profile, namely sensitivity toward all drugs tested except for ethambutol. Although both Mah strains were sensitive to the therapy administered, the dog's overall clinical status worsened progressively and the animal died 8 months after diagnosis. As far as we know, no resolution of disseminated MAC infections in dogs has been documented in the literature.

In conclusion, we report a rare case of disseminated mycobacteriosis in a crossbred dog where two distinct isolates of Mah were found. All the molecular typing approaches used, with the exception of 3′*hps65* sequencing, have proven to be useful tools for species and subspecies identification. Moreover, *rpsA* gene sequencing has made it possible to differentiate the Mah isolates into two distinct genotypic groups, making it a valuable means for discriminating *M. avium* below subspecies level.

## Data Availability Statement

All relevant data is contained within the article. The original contributions presented in the study are included in the article and in the Supplementary Material, further inquiries can be directed to the corresponding author.

## Ethics Statement

Not ethical approval was necessary because all laboratory investigations we described were performed to ensure the most effective therapy to the animal. No experiments have been carried out on the animal. Written informed consent was obtained from the owners for the participation of their animals in this study.

## Author Contributions

CM designed the study. CM, DA, and FR performed the experiments. CM analyzed the data and prepared the manuscript. CM, DA, and FR revised the manuscript. All authors contributed to the article and approved the submitted version.

## Conflict of Interest

The authors declare that the research was conducted in the absence of any commercial or financial relationships that could be construed as a potential conflict of interest.

## Supplementary Material

Supplementary Figure 1 Fine-needle aspiration cytology. Ziehl-Neelsen stain, 100x. Epithelioid macrophages containing numerous acid-fast bacilli, admixed with small lymphocytes. Bacteria are also in extracellular location in the background. The granulomatous in?ammatory response and acid-fast bacilli are suggestive of mycobacterial infection. Bar, 10 mm.

Supplementary Figure 2 Agarose gel showing the results of PCR assays for identification and differentiation of members of the genus *Mycobacterium*. Line 1: 100 bp DNA ladder; lines 2, 3, 4, 5, and 6: the primary broth culture, colonies 1 and 2, and *M. bovis* and *M. avium* field isolates, respectively, amplified for the genus of *Mycobacterium* (1030 bp); lines 7, 8, 9, 10, and 11: the primary broth culture, colonies 1 and 2, and *M. avium* and *M. bovis* field isolates, respectively, amplified for MTC (372 bp); lines 12, 13, 14, 15, and 16: the primary broth culture, colonies 1 and 2, and *M. avium* and *M. bovis* field isolates, respectively, amplified for *M. avium* (180 bp); lines 17, 18, 19, 20, and 21: the primary broth culture, colonies 1 and 2, and *M. avium* and *M. bovis* field isolates, respectively, amplified for *M. intracellulare*.
