# Supplementary figures and images for: Isolation, Molecular Typing, and Antibiotic Susceptibility Testing of Mycobacterium avium Subspecies hominissuis From a Dog With Generalized Mycobacteriosis
Source: Front Vet Sci. 2020 Nov 4;7:569966. doi: 10.3389/fvets.2020.569966 (PMC7672039; doi:10.3389/fvets.2020.569966)

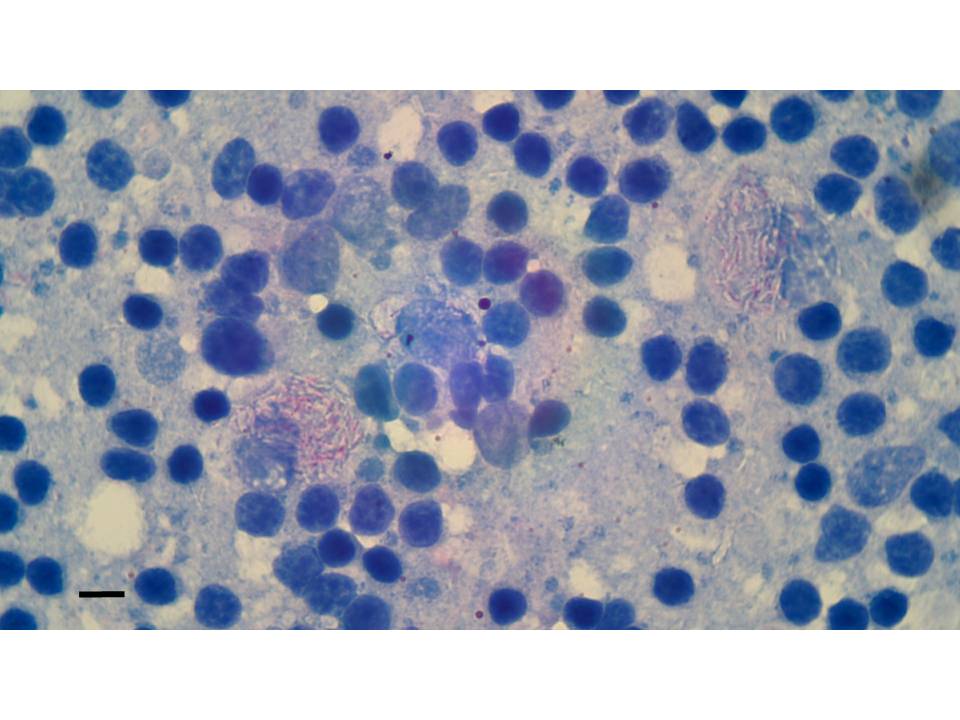

Supplement: Supplementary Figure 1 — Fine-needle aspiration cytology. Ziehl-Neelsen stain, 100x. Epithelioid macrophages containing numerous acid-fast bacilli, admixed with small lymphocytes. Bacteria are also in extracellular location in the background. The granulomatous in?ammatory response and acid-fast bacilli are suggestive of mycobacterial infection. Bar, 10 mm. [file Image_1.JPEG]

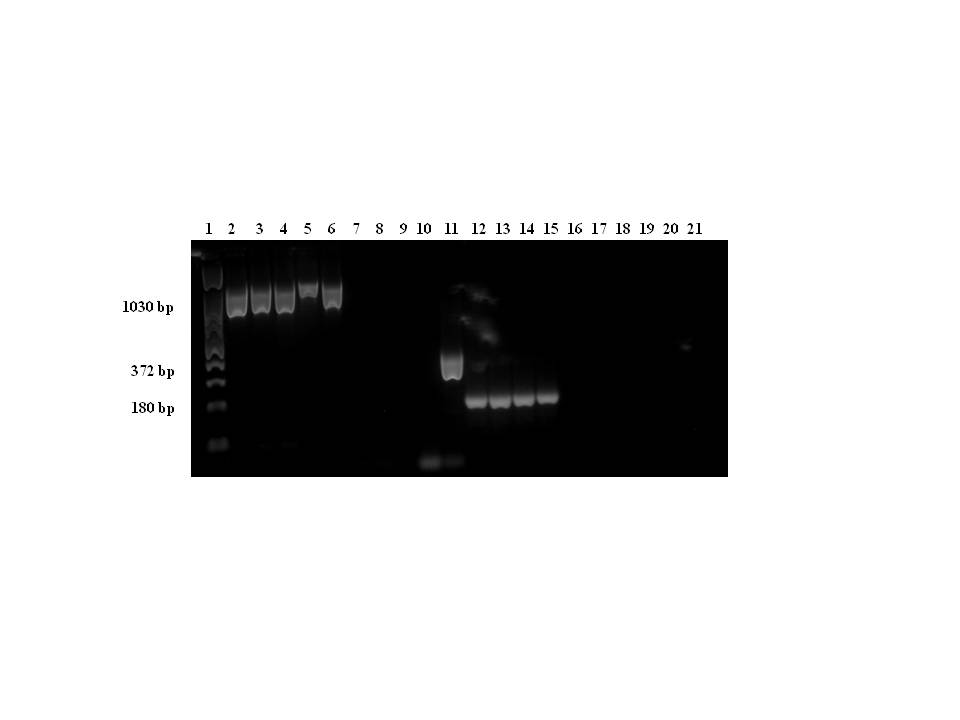

Supplement: Supplementary Figure 2 — Agarose gel showing the results of PCR assays for identification and differentiation of members of the genus Mycobacterium. Line 1: 100 bp DNA ladder; lines 2, 3, 4, 5, and 6: the primary broth culture, colonies 1 and 2, and M. bovis and M. avium field isolates, respectively, amplified for the genus of Mycobacterium (1030 bp); lines 7, 8, 9, 10, and 11: the primary broth culture, colonies 1 and 2, and M. avium and M. bovis field isolates, respectively, amplified for MTC (372 bp); lines 12, 13, 14, 15, and 16: the primary broth culture, colonies 1 and 2, and M. avium and M. bovis field isolates, respectively, amplified for M. avium (180 bp); lines 17, 18, 19, 20, and 21: the primary broth culture, colonies 1 and 2, and M. avium and M. bovis field isolates, respectively, amplified for M. intracellulare. [file Image_2.JPEG]
